# Test Performance and Potential Clinical Utility of the GenMark Dx ePlex Blood Culture Identification Gram-Negative Panel

**DOI:** 10.1128/spectrum.04092-22

**Published:** 2023-01-23

**Authors:** T. P. McCarty, P. Cumagun, J. Meeder, D. Moates, W. S. Edwards, J. Hutchinson, R. A. Lee, S. M. Leal

**Affiliations:** a Department of Medicine, Division of Infectious Diseases, The University of Alabama at Birmingham, Birmingham, Alabama, USA; b Department of Hospital Medicine, The University of Alabama at Birmingham, Birmingham, Alabama, USA; c UAB Fungal Reference Laboratory, Department of Pathology, Division of Laboratory Medicine, The University of Alabama at Birmingham, Birmingham, Alabama, USA; University of California, San Diego

**Keywords:** blood culture, Gram negative, molecular diagnostics

## Abstract

The test performance and potential clinical utility of the ePlex blood culture identification Gram-negative (BCID-GN) panel was evaluated relative to matrix-assisted laser desorption ionization–time of flight (MALDI-TOF) mass spectrometry on bacterial isolates and conventional antimicrobial susceptibility testing. The majority (106/108, 98.1%) of GN bacteria identified by MALDI were on the BCID-GN panel, and valid tests (107/108, 99.1%) yielded results on average 26.7 h earlier. For all valid tests with on-panel organisms, the positive percent agreement was 102/105 (97.2%) with 3 false negatives and the negative percent agreement was 105/105. Chart review (*n* = 98) showed that in conjunction with Gram stain results, negative pan-Gram-positive (GP) markers provided the opportunity to discontinue GP antibiotic coverage in 63/98 (64.3%) cases on average 26.2 h earlier. Only 8/12 (66.7%) *Enterobacterales* isolates with resistance to third-generation cephalosporins harbored the CTX-M gene. In contrast, 8/8 CTX-M^+^ samples yielded a resistant isolate. Detection of 1 Stenotrophomonas maltophilia (18 h), 1 OXA23/48^+^ Acinetobacter baumannii (52.4 h), and 3 CTX-M^+^ *Enterobacterales* isolates on ineffective treatment (47.1 h) and 1 on suboptimal therapy (72.6 h) would have additionally enabled early antimicrobial optimization in 6/98 (6.1%) patients.

**IMPORTANCE** The GenMark Dx ePlex rapid blood culture diagnostic system enables earlier time to identification of antimicrobial-resistant Gram-negative bacteria causing bloodstream infections. Its ability to rule out Gram-positive bacteria enabled early discontinuation of unnecessary antibiotics in 63/98 (64.3%) cases on average 26.2 h earlier. Detection of bacteria harboring the CTX-M gene as well as early identification of highly resistant bacteria such as Stenotrophomonas maltophilia and Acinetobacter baumannii enabled optimization of ineffective therapy in 6/98 (6.1%) patients. Its implementation in clinical microbiology laboratories optimizes therapy and improves patient care.

## INTRODUCTION

Gram-negative (GN) bacteremia is a major cause of morbidity and mortality (1, 2). Delayed administration of active antimicrobial agents correlates with poor outcomes, including death (3). Although broad-spectrum antimicrobial agents target many GN pathogens, this approach may not adequately treat bacteria with acquired or intrinsic resistance and is often suboptimal for infections with organisms susceptible to other antimicrobials (3). Rapid molecular identification of organisms and genes associated with specific resistance patterns direct from blood culture informs earlier escalation, deescalation, or discontinuation of antimicrobial agents (4–8). To exert an effect on patient care, rapid blood culture diagnostic results generated in the lab must be paired with antimicrobial stewardship and communication systems that rapidly inform front-line providers to act on these data-guided recommendations (8–15).

Several FDA-cleared commercial rapid blood culture diagnostic systems are currently available (5, 7, 16–24). The FDA-cleared GenMark blood culture identification Gram-negative (BCID-GN) panel includes a single-use cartridge which is inserted into 1 bay on the GenMark Dx ePlex instrument (25–30). The system is designed to rest on the benchtop, is scalable, and is random access, enabling simultaneous testing. It provides results in approximately 90 min for 21 bacteria and 6 antimicrobial resistance (AMR) genes including the CTX-M group of extended-spectrum β-lactamases (ESBL), carbapenemases (KPC, NDM, IMP, VIM, and OXA23/48), and highly resistant bacteria such as Stenotrophomonas maltophilia, Pseudomonas aeruginosa, and Acinetobacter baumannii, enabling early optimization/escalation of antimicrobial therapy (31–33). Detection of Neisseria meningitidis enables early contact tracing and antimicrobial chemoprophylaxis (34). Inclusion of Bacteroides fragilis complex and *Fusobacterium* spp. can prompt providers to maintain or add anaerobic coverage (35, 36). The inclusion of pan-Gram-positive (GP) targets increases provider confidence to discontinue GP antibiotic coverage.

In this study, we evaluated the performance of the BCID-GN panel compared to traditional standard of care (SOC) culture and susceptibility testing. Our data show high sensitivity and specificity for organism identification and detection of antimicrobial resistance genes. Additionally, we performed chart reviews and demonstrate significantly reduced time to organism identification and time to antimicrobial resistance (AMR) gene detection, potentially enabling more rapid optimization or discontinuation of antimicrobial therapy when included in the routine workflow of blood cultures at our institution.

## RESULTS

### Test performance characteristics—organism identification.

Table 1 highlights the overall performance of the BCID-GN panel to identify GN bacteria relative to traditional culture and matrix-assisted laser desorption ionization–time of flight mass spectrometry (MALDI-TOF MS). A total of 108 positive blood culture bottles were evaluated, and most tests, 107/108 (99.1%), yielded valid results. Only 2 organisms (106/108; 98.1%) not represented on the panel (*Providencia* and *Veillonella*) grew in culture. One sample with Pantoea dispersa failed to amplify the internal control after two attempts and was excluded from test performance characteristic analyses. Overall, the BCID-GN panel exhibited a positive percent agreement (PPA) of 102/105 (97.2%) and negative percent agreement (NPA) of 105/105 (100%) with 3 false negatives (1 Escherichia coli, 1 Klebsiella aerogenes, 1 Klebsiella pneumoniae) based on organism growth in culture. E. coli (*n* = 34), K. pneumoniae (*n* = 28), P. aeruginosa (*n* = 14), and Enterobacter species (*n* = 9) were the most frequently isolated organisms. Coinfections with multiple GN bacteria (7/105; 6.7%) were noted as well as mixed infections with GP bacteria (6/105; 5.7%) including 2 Enterococcus faecium, 1 Enterococcus faecalis, and 1 Staphylococcus aureus isolate likely representing true infection and 1 Streptococcus cristatus and 1 Streptococcus gallolyticus isolate possibly representing contamination. No mixed infections with fungi were identified.

**TABLE 1:** Sensitivity/PPA and specificity/NPA of organism ID by the BCID-GN panel versus MALDI-TOF MS

Bacterium(-a) identified by MALDI in each positive blood culture	Total	Sensitivity/PPA		Specificity/NPA	
		No. of TP/no. of TP+FN	% (95% CI)	No. of TN/no. of TN+FP	% (95% CI)
Acinetobacter baumannii	3	3/3	100 (29.3–100)	102/102	100 (96.5–100)
Acinetobacter baumannii, Enterococcus faecalis^*a*^	1	1/1	100 (2.5–100)	104/104	100 (96.5–100)
Citrobacter freundii	2	2/2	100 (15.8–100)	103/103	100 (96.5–100)
Citrobacter werkmanii, Citrobacter youngae	1	1/1	100 (2.5–100)	104/104	100 (96.5–100)
Enterobacter cloacae complex	7	7/7	100 (59.0–100)	98/98	100 (96.3–100)
Enterobacter cloacae, Klebsiella pneumoniae	1	1/1	100 (2.5–100)	104/104	100 (96.5–100)
Enterobacter cloacae, Pseudomonas aeruginosa	1	1/1	100 (2.5–100)	104/104	100 (96.5–100)
Escherichia coli ^*b*^	34	33/34	97.1 (84.7–99.9)	71/71	100 (95.0–100)
Escherichia coli and Streptococcus gallolyticus^*a*^	1	1/1	100 (2.5–100)	104/104	100 (96.5–100)
Klebsiella (Enterobacter) *aerogenes*^*b*^	2	1/2	50 (1.3–98.8)	103/103	100 (96.5–100)
Klebsiella oxytoca	4	4/4	100 (39.8–100)	101/101	100 (96.4–100)
Klebsiella oxytoca, Citrobacter freundii	1	1/1	100 (2.5–100)	104/104	100 (96.5–100)
Klebsiella pneumonia, Escherichia coli	2	2/2	100 (15.8–100)	103/103	100 (96.5–100)
Klebsiella pneumoniae ^*b*^	22	21/22	100 (77.2–99.9)	83/83	100 (95.7–100)
Klebsiella pneumoniae, Enterococcus faecium^*a*^	1	1/1	100 (2.5–100)	104/104	100 (96.5–100)
Klebsiella pneumoniae, Streptococcus cristatus^*a*^	1	1/1	100 (2.5–100)	104/104	100 (96.5–100)
Klebsiella pneumonia, Staphylococcus aureus^*a*^	1	1/1	100 (2.5–100)	104/104	100 (96.5–100)
Pantoea dispersa ^*c*^	1	1/1	100 (2.5–100)	104/104	100 (96.5–100)
Proteus mirabilis	2	2/2	100 (15.8–100)	103/103	100 (96.5–100)
Providencia stuartii ^*d*^	1	1/1	100 (2.5–100)	104/104	100 (96.5–100)
Pseudomonas aeruginosa, Enterococcus faecium^*a*^	1	1/1	100 (2.5–100)	104/104	100 (96.5–100)
Pseudomonas aeruginosa	11	11/11	100 (71.5–100)	94/94	100 (96.2–100)
Salmonella spp.	1	1/1	100 (2.5–100)	104/104	100 (96.5–100)
Serratia marcescens	3	3/3	100 (29.3–100)	102/102	100 (96.5–100)
Serratia marcescens, Pseudomonas aeruginosa	1	1/1	100 (2.5–100)	104/104	100 (96.5–100)
Stenotrophomonas maltophilia	1	1/1	100 (2.5–100)	104/104	100 (96.5–100)
*Veillonella* ^*d*^	1	1/1	100 (2.5–100)	104/104	100 (96.5–100)
Overall performance	108	102/105^*c*^^,^^*d*^	97.2 (91.9–99.4)	105/105	100 (96.6–100)

a Indicates samples with mixed GP and GN bacteria; all 6 triggered the pan-GP target.

b BCID-GN did not detect 3 microbes (1 E. coli, 1 K. aerogenes, and 1 K. pneumoniae isolate).

c Amplification of internal controls failed for 1 sample (Pantoea dispersa).

d Organisms not included on the BCID-GN panel (1 *Providencia* and 1 *Veillonella* isolate).

### Test performance characteristics—antimicrobial resistance.

Table 2 highlights the overall performance of the BCID-GN panel to identify antimicrobial resistance relative to traditional antimicrobial susceptibility testing (AST). A total of 8 samples with *Enterobacterales* isolates including 5 E. coli and 3 K. pneumoniae tested positive for the CTX-M gene, and all of them exhibited resistance to third-generation cephalosporins, resulting in a PPA of 8/8 (100%) and NPA of 97/97 (100%). Consistent with alternative resistance mechanisms, only 8/12 (66.7%) *Enterobacterales* isolates with resistance to third-generation cephalosporins harbored the CTX-M gene. The gene encoding OXA23/48 was detected in a single sample harboring carbapenem-resistant A. baumannii. S. maltophilia was also detected in a single sample.

**TABLE 2:** Opportunities to optimize antimicrobials based on the BCID-GN panel result

Opportunity to optimize antimicrobials	Total	Change	Sensitivity/PPA		Specificity/NPA	
			No. of TP/no. of TP+FN	% (95% CI)	No. of TN/no. of TN+FP	% (95% CI)
CTX-M (5 E. coli, 3 K. pneumoniae isolates)^*a*^	8	Optimize	8/8	100 (63.0–100)	97/97	100 (96.3–100)
OXA23/48 (A. baumannii)	1	Escalate	1/1	100 (2.5–100)	104/104	100 (96.5–100)
S. maltophilia	1	TMP/SMX	NA^*b*^	NA	NA	NA
Total	10	9.3% (10/108) of tests yielded potentially clinically actionable results				

a Consistent with alternative resistance mechanisms, only 8/12 (66.7%) *Enterobacterales* isolates with resistance to third-generation cephalosporins harbored the CTX-M gene.

b NA, not available.

### Patient demographics and potential clinical utility.

Table 3 summarizes patient demographics and comorbidities including the presence of an immunocompromising condition. The most common comorbidities in this cohort were diabetes (30.6%), chronic lung disease (20.4%), and cardiovascular disease (16.3%). Immunosuppression was very common, comprising 51% of patients. Overall, empirical therapy varied significantly in this cohort, encompassing 26 different regimens with only 1/98 (1.0%) patients not receiving empirical antibiotics, 32/98 (32.7%) receiving a single antibiotic, and 22/98 (22.4%) on antipseudomonal coverage. Most patients receiving multiple antibiotics (65/98; 66.3%) received both antipseudomonal (60/65; 92.3%) and anti-MRSA (methicillin-resistant S. aureus) coverage (62/65; 95.4%).

**TABLE 3:** Patient demographics and comorbidities

Variable^*a*^	Total (*n* = 98)
Age (yr)	
Mean	58.9
Median	61.5
Range	22–95
Male, no. (%)	57 (58.1)
Race/ethnicity, no. (%)	
White	57 (58.2)
Black	40 (40.8)
Asian	1 (1.0)
Immunosuppression, no. (%)	
Solid malignancy	15 (15.3)
Hematologic malignancy	6 (6.1)
SOT	9 (9.2)
HSCT	3 (3.1)
Other	17 (17.4)
Diabetes, no. (%)	30 (30.6)
Cardiovascular disease, no. (%)	16 (16.3)
Chronic lung disease, no. (%)	20 (20.4)
CKD, no. (%)	16 (16.3)
ESRD, no. (%)	6 (6.1)
Cirrhosis, no. (%)	13 (13.3)
IVDU, no. (%)	3 (3.1)
Mechanical ventilation, no. (%)	18 (18.4)
Trauma at time of admission, no. (%)	9 (9.2)
Burn at time of admission, no. (%)	1 (1.0)
Pitt bacteremia score (mean)	2.7

a SOT, solid organ transplant; HSCT, hematopoietic stem cell transplant; CKD, chronic kidney disease; ESRD, end-stage renal disease; IVDU, intravenous drug use.

Table 4 highlights the potential opportunities for antimicrobial optimization identified upon chart review (*n* = 98). In conjunction with the Gram stain (GS), 49/98 (50%) samples with negative pan-GP-marker results could have enhanced provider confidence to discontinue unnecessary GP coverage 26.2 h earlier than SOC methods. In contrast, codetection of GP bacteria in 6/98 (6.1%) samples could have helped ensure that providers maintained dual coverage. Of the 8 CTX-M^+^ cases, 3/98 (3.1%) were on ineffective therapy (piperacillin-tazobactam, ceftriaxone, or ciprofloxacin), which could have been optimized 47.1 h earlier. An additional patient (1/98; 1.0%) infected with a CTX-M^+^ *Enterobacterales* isolate was on suboptimal ceftazidime-avibactam monotherapy and could have been deescalated to a carbapenem 72.6 h earlier. One A. baumannii isolate expressing OXA23/48 and 1 S. maltophilia isolate (both 1/98; 1.0%) could have prompted earlier escalation of antibiotic therapy an average of 52.4 h and 18.0 h, respectively.

**TABLE 4:** Type of change to antibiotic regimen and time saved based on BCID-GN results

Test result	No. of patients affected/total no. (%)	Action	Mean time saved (h)
Negative pan-GP-marker data	49/98 (50)	Remove GP coverage^*a*^	26.2
Coinfection with GP bacteria	6/98 (6.1)	Maintain dual coverage	NA^*c*^
CTX-M on ineffective therapy^*b*^	3/98 (3.1)	Escalate antibiotic	47.1
CTX-M on suboptimal therapy^*b*^	1/98 (1.0)	Deescalate antibiotic	72.6
Carbapenemase gene (OXA23/48)	1/98 (1.0)	Escalate antibiotic	52.4
Stenotrophomonas maltophilia	1/98 (1.0)	Escalate antibiotic	18.0

a In conjunction with GS, negative pan-GP-marker data could have helped providers make the decision to remove GP antibiotic coverage.

b Ineffective antibiotics included piperacillin-tazobactam, ceftriaxone, and ciprofloxacin. Ceftazidime-avibactam was deemed a suboptimal therapeutic option.

c NA, not available.

## DISCUSSION

In this study, the BCID-GN test exhibited a PPA of 102/105 (97.2%) and an NPA of 105/105 (100%). This performance is in agreement with the 96% PPA reported by both Huang et al. in Belgium and Bryant et al. in France and exceeds the overall accuracy of 89.7% PPA observed by Krifors et al. in Sweden and the 92.9% observed in a U.S. multicenter trial (25, 27, 28, 30). Although our patient population is ≥18 years old, our data are also comparable to the 93% overall accuracy observed in a pediatric oncology population (37). Although a subset of these prior studies utilized research-use-only (RUO) test cartridges, there is no difference in design or production with the FDA-cleared *in vitro* diagnostic (IVD) cartridges utilized in the current study (25, 27, 28, 30). The 3 false-negative organisms include 2 pansusceptible *Enterobacterales* isolates (K. aerogenes and K. pneumoniae) and 1 extended-spectrum β-lactamase (ESBL) E. coli isolate. No change in treatment would have been recommended if the K. aerogenes or K. pneumoniae isolate had been detected. Sequencing of the E. coli isolate identified it as a known rare strain carrying sequence mutations at a key oligonucleotide binding site, and future versions of this test are expected to address this known issue. This patient was already being treated with meropenem, and since AMR genes were not detected, it is not clear if species-level identification on its own would have prompted a change in therapy.

The PPA for organisms on the BCID-GP panel is comparable to that of other FDA-approved rapid blood culture diagnostic systems including the Verigene (Luminex) and FilmArray (bioMérieux) (17, 38). However, the BCID-GN panel includes more organisms than comparator systems, enabling broad coverage. Relative to the BioFire BCID2, the ePlex BCID GN additionally detects *Citrobacter*, Cronobacter sakazakii, Enterobacter (non-*cloacae* complex), Fusobacterium nucleatum, Fusobacterium necrophorum, Morganella morganii, Proteus mirabilis, and *Serratia*. Relative to the Verigene BC-GN, the ePlex BCID-GN detects all the organisms listed above as well as A. baumannii, Bacteroides fragilis, Enterobacter cloacae complex, Haemophilus influenzae, Neisseria meningitidis, Proteus mirabilis, Salmonella, Serratia marcescens, and S. maltophilia. Utilizing the sample cohort in the current study, only 3/108 (2.7%) of total blood cultures contained a GN organism not represented on the BCID-GN panel including *Veillonella*, *Pantoea*, and *Providencia* species. In contrast, 13/108 (12.0%) and 27/108 (25%) of samples in this study contained organisms not targeted by the BioFire BCID2 or Verigene BC-GN test, respectively.

All 3 systems detect CTX-M and the 5 major carbapenemase genes, but only the BioFire BCID2 panel additionally detects the *mcr1* gene, conferring colistin resistance (17, 39). Although colistin resistance is currently rare in the United States, it is an increasingly important emerging global health threat (40). In this study, the detection of 1 S. maltophilia isolate, 1 A. baumannii isolate expressing OXA23/48, and 4 *Enterobacterales* isolates expressing CTX-M represented opportunities for early optimization of antimicrobial therapy in 6/98 (6.1%) samples. Krifors et al. in Sweden identified 3/29 (10.3%; 1 CTX-M, 2 B. fragilis) and Huang et al. in Belgium detected 14/122 (11.5%; 10 CTX-M, 4 B. fragilis) cultures as potentially clinically actionable (27, 28). However, Bryant et al. in France identified a 2-fold increase in clinically actionable cultures (22/107; 20.6%; 17 CTX-M, 5 B. fragilis) (25). This difference in clinical actionability is likely a consequence of variable antimicrobial resistance rates in patient populations and geographic areas (31). The ability of rapid blood culture diagnostic systems to make an increasingly greater impact as a function of resistance rates in the population is impressive and argues strongly for dissemination of this technology to underserved communities and geographic areas in which resistance rates are highest.

In this study, 8/8 isolates from samples harboring the CTX-M gene were resistant to third-generation cephalosporins. These data are consistent with high sensitivity and specificity for CTX-M reported in the literature (26, 27, 30, 31, 37). However, consistent with alternative resistance mechanisms, only 8/12 (66.7%) *Enterobacterales* isolates with resistance to third-generation cephalosporins harbored the CTX-M gene, indicating that although a positive CTX-M result should prompt consideration of therapy escalation, a negative result is insufficient evidence to support deescalation.

The prevalence of the CTX-M gene in 8/98 (8.2%) total samples is less than the 11% observed in a recent study of 66 U.S. hospitals by Tamma et al. and significantly lower than the high rates of 22 to 26% noted in certain geographic areas (30, 31). Similar to the findings of Tamma et al., both E. coli and K. pneumoniae were the most common CTX-M-expressing species isolated, with a prevalence in E. coli of 5/37 (13.5%; compared to 16%) and K. pneumoniae of 3/28 (10.7%; compared to 14%) (31). BCID-GN testing in geographic hot spots with high CTX-M prevalence should exhibit significantly greater rates of clinically actionable test results than the current study.

The detection of 1 A. baumannii isolate expressing OXA23/48 enabled rapid escalation of antimicrobial therapy (32). Carbapenemase genes were not detected in recent evaluations by Bryant et al. in France (*n* = 107), Huang et al. in Belgium (*n* = 122), or Krifors et al. in Sweden (*n* = 29) (25, 27, 28). A recent U.S.-based multicenter BCID-GN study detected 17 clinical samples with carbapenemase genes (7 KPC; 10 OXA23/48); however, as the scope of the study was test performance, not epidemiology, repeat cultures from known patients were included (30). Our data are consistent with those of Tamma et al., who recently detected carbapenemase genes in only 61/4,209 (1.5%) positive blood culture bottles and identified A. baumannii as the most common (5%) carbapenem-expressing organism in these samples (31).

Detection of 18/105 (18.1%) species known to commonly harbor high levels of antimicrobial resistance (4 A. baumannii, 14 P. aeruginosa, and 1 S. maltophilia isolate) helps providers confidently escalate therapy to include multidrug-resistant (MDR) pathogens (41, 42). Early detection of S. maltophilia specifically enables the addition of trimethoprim-sulfamethoxazole (TMP/SMX) (43). The clinical utility of rapidly detecting MDR pathogens in blood additionally increases with the medical complexity, carriage rates, and exposure of the patient population to these environmental glucose nonfermenters (e.g., cystic fibrosis patients, burn units, chronic wounds, etc.).

Although MALDI-TOF MS methods can detect a broader range of organisms directly from positive blood culture bottles, they do not work well for mixed infections and would not have yielded a result in 13/105 (12.4%) samples in this cohort (44–47). Likewise, although MALDI-TOF MS directly from blood would have enabled the rapid identification of S. maltophilia, this method does not currently detect antimicrobial resistance or AMR gene expression and would not have detected CTX-M expression in the 8 *Enterobacterales* isolates or OXA23/48 in the single A. baumannii isolate, yielding a very low predicted clinical actionability rate of 1/98 (1.0%).

The inclusion of pan-targets provides a rapid backup for misinterpreted Gram stains and aids in the interpretation of slides with Gram-variable organisms or antibiotic effect (48, 49). In this study, coinfections with multiple GN bacteria (7/105; 6.7%) were noted as well as mixed infections with GP bacteria (6/105; 5.7%) including 2 Enterococcus faecium, 1 E. faecalis, and 1 S. aureus isolate likely representing true infection and 1 Streptococcus cristatus and 1 S. gallolyticus isolate possibly representing contamination. No mixed infections with fungi were identified. In 3/6 (50%) Gram stain (GS) interpretations, the GP organism was not noted despite growth in culture. This high rate may be due to decreased sensitivity of the GS relative to the BCID pan-target and/or the predominance of fast-growing GN bacteria at the time of the stain. In this study, the GP organisms that were not reported on the initial GS included 1 vancomycin-sensitive enterococcus (VSE) E. faecalis in an A. baumannii coinfection, 1 vancomycin-resistant enterococcus (VRE) E. faecalis in a P. aeruginosa coinfection, and 1 methicillin-susceptible S. aureus (MSSA) in a K. pneumoniae coinfection. Backup detection with the BCID-GN pan-GP marker in these 3 scenarios would have rapidly corrected this interpretation error and possibly helped mitigate inappropriate discontinuation of GP antimicrobial coverage. Additionally, once implemented into the lab workflow, the pan-markers continually reinforce the need for staff to perform thorough GS reviews because ~90 min later the molecular result is available for comparison, enabling real-time personal and/or supervisor feedback. Lastly, given the high degree of empirical combination therapy in patients, the high rate of pan-GP-marker negative results observed would have provided increased certainty to treating clinicians to discontinue unnecessary and potentially harmful GP antibiotic coverage.

In contrast to GP bacterial infections, there is less predictability in translating the lack of an AMR gene to *in vitro* susceptibility results, given the greater degree of diversity in resistance mechanisms. A combination of organism identification, absence of AMR genes, and utilization of a local antibiogram could enable much earlier optimization of antibiotics but should be done in conjunction with the expertise of antimicrobial stewardship or infectious disease consultation. While stewardship is traditionally seen as synonymous with narrowing, it is important to note the potential for earlier recognition of resistance and optimization of antimicrobial therapy as seen in the 6/98 (6.1%) patients receiving ineffective antibiotics found to have either a resistant species or genetic resistance present.

Although rapid identification of anaerobic *Fusobacterium* species and B. fragilis can prompt the addition of anaerobic coverage to empirical antimicrobial regimens, there were no on-panel anaerobes in our sample cohort (35, 36). Likewise, although rapid detection of H. influenzae and N. meningitidis enables earlier reporting to state public health laboratories, contact tracing, and chemoprophylaxis, these organisms were not detected in our cohort (34).

In conclusion, the ePlex BCID-GN panel provides rapid organism identification and AMR gene detection with high accuracy compared to traditional culture and MALDI-TOF MS on bacterial isolates. The broad panel and unique inclusion of MDR pathogens, anaerobes, reportable organisms, pan-markers, and AMR genes, including CTX-M and the 5 most common carbapenemase genes, yields clinically actionable results dependent on the prevalence of AMR-gene dependent antimicrobial resistance in the patient population. Additional studies evaluating patient outcomes before and after implementation of the BCID-GN panel are needed to determine more accurately the real-world clinical and cost-savings impact of this rapid blood culture diagnostic system.

## MATERIALS AND METHODS

### Sample collection and processing.

**FIG 1:**
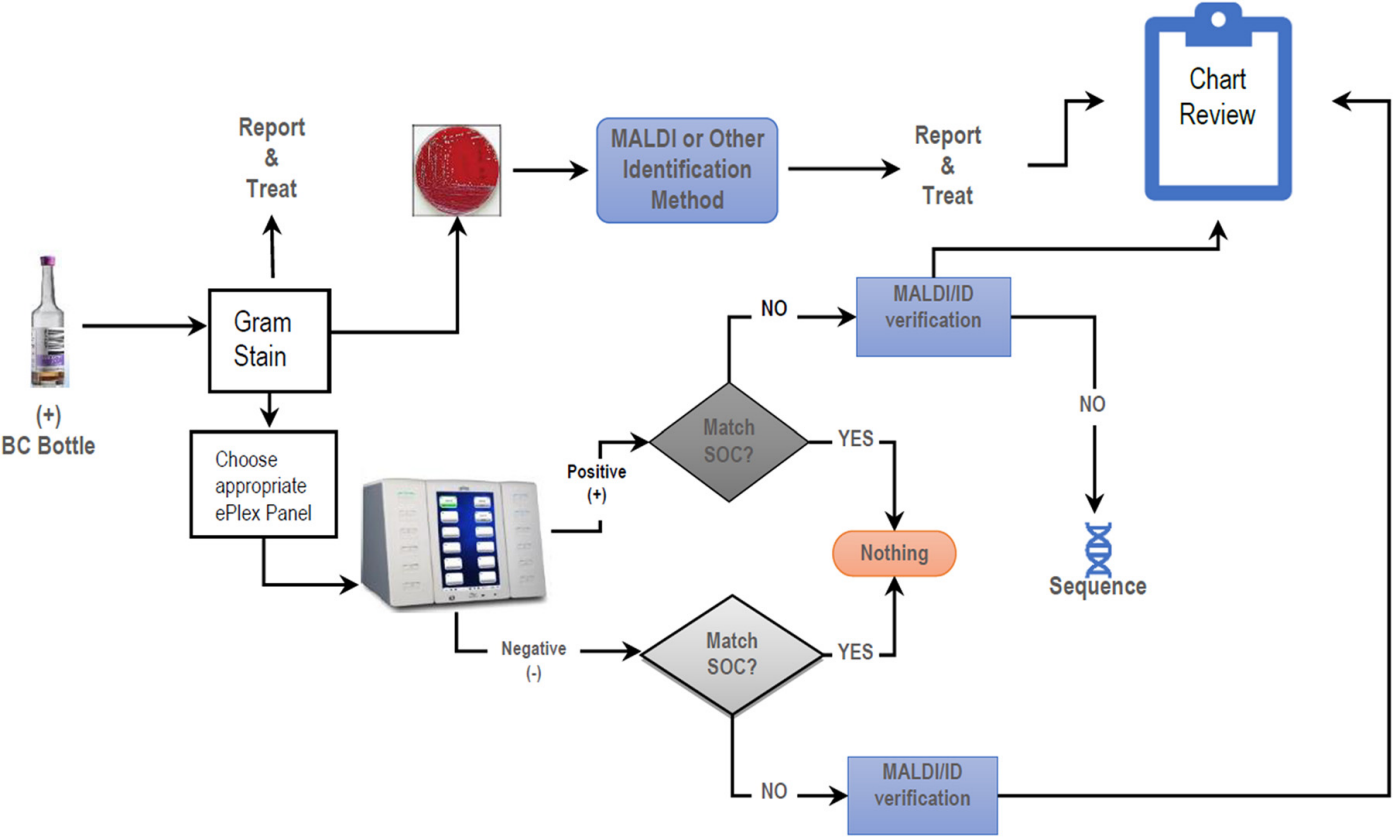
Study design. Positive blood culture (BC) bottles received standard of care Gram stain, culture, and identification of isolated colonies using Vitek MS MALDI-TOF mass spectrometry. Within 48 h, positive blood culture bottles were also evaluated by the study technologist using the BCID-GN panel on the GenMark ePlex system. Discordant identifications were evaluated by repeat MALDI-TOF MS and/or 16S sequencing, and chart reviews were performed to determine potential clinical utility.

Figure 1 highlights the study design utilized to determine the test performance characteristics and potential clinical utility of the GenMark ePlex BCID-GN panel compared to traditional SOC culture and susceptibility testing. Peripheral blood was collected via standard venipuncture into bioMérieux BacT/Alert resin FA/FN blood culture bottles and cultured on the BacT/Alert Virtuo automated blood culture system. Aliquots of positive blood cultures with GN bacteria detected on Gram stain (GS) (*n* = 108) received SOC culture and antimicrobial susceptibility testing (AST). Identification of bacterial isolates on solid medium was performed utilizing the FDA-cleared bioMérieux Vitek MS matrix-assisted laser desorption ionization (MALDI)–time of flight mass spectrometry system with software version 3.2.0. Susceptibility testing was performed utilizing the Siemens MicroScan and/or standard antimicrobial disk diffusion and gradient diffusion. After completion of standard of care processing, all positive blood culture bottles in the clinical microbiology lab were stored in a designated bin at room temperature (≤48 h), at which time the study technologist chose a random subset (not consecutively) to perform the BCID-GN panel. If the original run failed (internal control not detected), the test was repeated. If the pan-Gram-positive or panfungal markers were detected, the sample was additionally evaluated on the BCID-GP or BCID-fungal panel, respectively.

### Chart review.

Only SOC results (e.g., MALDI identifications) were reported into the electronic medical record (EMR) (Cerner PowerChart) and available to inform clinical decisions. Providers did not have access to BCID-GN panel results. Chart reviews (*n* = 98) were performed by infectious disease clinicians in order to evaluate the potential impact of BCID-GN panel results on the time to organism identification, AST results, and optimization of antimicrobial therapy. Additional data collected included demographics, medical comorbidities, assessment of true infection versus contamination, antibiotic usage, length of stay, and mortality. The following cultures were excluded from the analysis: subsequent positive cultures rather than initial positive cultures (*n* = 5), positive cultures harboring a bacterial species not on the panel (*n* = 3), positive blood cultures from an organ procurement (*n* = 1), and age under 18 years (*n* = 1). True pathogens were noted if GN bacteria were identified in one or more culture bottles. Skin flora identified in only one of several blood culture sets were defined as contaminants upon clinical review by two infectious disease physicians (P.C. and R.A.L.). We defined antimicrobial optimization as improvement in a selected regimen that is both effective and appropriate to treat the infection of the individual patient while minimizing the development of drug resistance; this may include escalation or deescalation of antibiotics depending on the pathogen. Potential cost savings associated with escalation, deescalation, and discontinuation of antibiotics were calculated utilizing University of Alabama at Birmingham (UAB) pharmacy drug costs and patient-specific time intervals between the availability of BCID-GN results (set at 2 h after GS result entry into the EMR) and the corresponding organism identification (ID) or susceptibility result (both documented in EMR) that prompted the therapeutic change. This study was approved by the UAB Institutional Review Board, study number 300003729.

### Discordant analyses.

BCID-GN panel results were deemed false negative if the assay failed to detect an organism or antimicrobial resistance pattern identified by comparator methods. A false positive BCID-GN result was noted if an organism or AMR gene was identified on the panel but not the comparator method. Discordant identifications were adjudicated via repeat culture and MALDI-TOF MS, reevaluation of GS morphology, and/or 16S sequencing of isolates and blood culture aliquots. Adjudication of discordant resistance patterns was performed by repeat testing with disk or gradient diffusion.

### Statistical methods.

Positive percent agreement (PPA) and negative percent agreement (NPA) were determined for all samples included in the study (*n* = 105) and for each blood culture from which a single organism or unique collection of microbes was isolated. PPA was calculated as 100 × number of TP/(number of TP + number of FN), and NPA was calculated as 100 × number of TN/(number of TN + number of FP), where TP is true positives, FN is false negatives, TN is true negatives, and FP is false positives. The two-sided 95% score confidence interval (CI) was calculated for PPA and NPA. The Clopper-Pearson method was used to calculate 95% confidence intervals for each test performance characteristic.
